# A GRAPHICAL MODEL APPROACH TO UNVEILING FALL TRIGGERS IN COMMUNITY-DWELLING OLDER ADULTS

**DOI:** 10.1093/geroni/igae098.3359

**Published:** 2024-12-31

**Authors:** Tho Nguyen, Chang Liu, Yi Liu, Ladda Thiamwong, Qian Lou, Rui Xie

**Affiliations:** University of Central Florida, Orlando, Florida, United States; University of Central Florida, Orlando, Florida, United States; University of Central Florida, Orlando, Florida, United States; University of Central Florida, Orlando, Florida, United States; University of Central Florida, Orlando, Florida, United States; University of Central Florida, Orlando, Florida, United States

## Abstract

We aim to enhance understanding of fall risk factors and examine their interrelationships in community-dwelling older adults from various domains: sociodemographics, mental well-being, body composition, self-assessed and performance-based fall risk assessments, and physical activity patterns. Our study included 120 subjects aged 60 to 96 years old from Central Florida (average age of 74.8, SD 7.38), 77.5% female, and 29.2% with a history of falling. We used a novel machine learning technique known as the Graphical Model (GM) to analyze the association among 37 fall risk factors. GM reveals the higher-order dependence structure and graphical relationships among variables, including continuous and categorical types. This approach goes beyond traditional correlation analysis, providing innovative insights into sophisticated interactions within a high-dimensional dataset. Among the identified features, 34 exhibited pairwise relationships, while COVID-19-related factors and housing composition remained conditionally independent. Prominent relationships include sedentary behavior having strong negative links to both light physical activity and moderate-to-vigorous physical activity. Participant age was positively correlated with balance score but negatively correlated with body composition factors and Senior Technology Acceptance. The CDC’s Stopping Elderly Accidents, Deaths, and Injuries score had moderate positive relationships with the Rapid Assessment of Physical Activity (strength and flexibility) and Mindful Attention Awareness Scale, while it had moderate negative relationships with the Short Falls Efficacy Scale-International score and history of falls. The findings of this study serve as an initial exploration, laying the foundation for future research, including the longitudinal dynamics of these features that contribute to fall prevention in older adults.

